# Navigating the challenges of minimally invasive mitral valve surgery: a risk analysis and learning curve evaluation

**DOI:** 10.1186/s13019-024-02479-3

**Published:** 2024-01-23

**Authors:** Nestoras Papadopoulos, Vasileios Ntinopoulos, Stak Dushaj, Achim Häussler, Dragan Odavic, Hector Rodríguez Cetina Biefer, Omer Dzemali

**Affiliations:** 1 Department of Cardiac Surgery, City Hospital, Birmensdorferstrasse 497, 8063 Zurich, Switzerland; 2 Department of Cardiac Surgery, University Hospital Zürich, Zurich, Switzerland; 3 Department of Cardiology, Center of Experimental and Translational Cardiology (CTEC), University Hospital of Zurich, University of Zurich, Zurich, Switzerland

**Keywords:** Surgical education, Minimally invasive cardiac surgery, Mitral valve surgery, Mitral valve reconstruction

## Abstract

**Background:**

This study aimed to report the risk and learning curve analysis of a minimally invasive mitral valve surgery program performed through a right mini-thoracotomy at a single institution.

**Methods:**

From January 2013 through December 2019, 266 consecutive patients underwent minimally invasive mitral valve surgery in our department and were included in the current study. Multiple logistic regression analysis was used for the adverse event outcome. Distribution over time of perioperative complications, defined as clinical endpoints in the Valve Academic Research Consortium-2 (VARC-2) consensus document, as well as CUSUM charts for assessment of cardiopulmonary bypass and aortic cross-clamping duration over time, has been performed for learning curve assessment.

**Results:**

Overall incidences of postoperative stroke (1.1%), myocardial infarction (1.1%), and thirty-day mortality (1.5%) were low. The mitral valve reconstruction rate in our series was 95%. Multivariable analysis revealed that concomitant tricuspid valve surgery (OR 4.44; 95%CI 1.61–11.80; *p* = 0.003) was significantly associated with adverse event outcomes. Despite a trend towards adverse event outcomes in patients with preexisting active mitral valve endocarditis (OR 2.69; 95%CI 0.81–7.87; *p* = 0.082), mitral valve pathology did not significantly impact postoperative morbidity and mortality. Distribution over time of perioperative complications, defined as clinical endpoints in the VARC-2 consensus document, showed a trend towards an improved complication rate after the initial 65–100 procedures.

**Conclusions:**

Mitral valve surgery via right-sided mini-thoracotomy can be implemented safely with low perioperative morbidity and mortality rates. Careful patient selection regarding isolated mitral valve surgery in the presence of degenerative mitral valve disease may represent a significant safety issue during the learning curve.

*Trial Registration*: The cantonal ethics commission of Zurich approved the study (registration ID 2020-00752, date of approval 24 April 2020).

## Background

Minimally invasive mitral valve surgery (MIV-MVS) through right mini-thoracotomy represents an excellent surgical route for mitral valve surgery [1, 2]. Preserving the integrity of the sternum, esthetics, and faster convalescence lead to increased patient demand for the minimally invasive mitral valve approach [3]. Thus, the interest in implementing a minimally invasive mitral valve program in several departments for cardiothoracic surgery has increased exponentially in the last decade [4].

The main concern in that field remains the risk of a learning curve hampering the growth of MIV-MVS. Initial patient selection and the number of patients needed to overcome a substantial learning curve are still controversial discussed in the current literature [5, 6].

In 2013, we initiated a minimally invasive mitral valve surgery program in our institution. In the current manuscript, we aim to report our experience and lessons learned from implementing MIV-MVS through a right mini-thoracotomy in the modern era, assessing our learning curve under evaluation of clinical and echocardiographic midterm follow-up data. Finally, an assessment of risk factors associated with adverse outcomes during the establishment of our minimally invasive mitral valve surgery program has been performed.

## Methods

This single-center study includes patients undergoing mitral valve procedures through a right mini-thoracotomy between January 2013 and December 2019 at the City Hospital of Zurich, Switzerland. All of the procedures were performed by a single leading surgeon with significant experience in mitral valve surgery via sternotomy. First, operations were performed on elective cases of isolated mitral valve insufficiency. In the first year of our knowledge (2013), mitral valve endocarditis and urgent or emergency mitral valve procedures were considered contraindications for a minimally invasive approach. Since 2014, all mitral valve procedures independent of the underlying pathology or urgency were performed via lateral mini-thoracotomy. The first Barlow mitral valve was treated through a minimally invasive approach in 2014. The first concomitant procedure was also performed in 2014. For this study, we considered 266 consecutive patients with mitral valve pathology as a primary indication for surgery. Concomitant procedures were performed in 98 patients: tricuspid valve repair, ablation, atrial septal defect correction, and left atrial appendage closure. Patients operated on through a right mini-thoracotomy with a primary indication for atrial myxoma and isolated tricuspid valve pathology were not included in the current study. Contraindications for the performance of minimally invasive accessway in our series were pectus excavatum, previous surgical procedures in the right thoracic cavity, and concomitant moderate to severe aortic valve regurgitation. Demographic, preoperative, intraoperative, and postoperative data were obtained from the prospectively entered data of the database of our clinic (Dendrite Clinical Systems Ltd, Reading, UK).

### Follow-up

All patients were examined with transthoracic echocardiography before discharge for cardiac rehabilitation. Follow-up was performed by contacting the referring cardiologist to obtain the latest echocardiographic examination. In case of missing data, additional information was acquired through the family physician or by phone contact with patients and family members. A transthoracic echocardiography report was obtained for 245 patients. Thus, the echocardiographic follow-up was 97% complete.

### Ethics statement

The cantonal ethics commission of Zurich approved the study (registration ID 2020-00752, date of approval 24 April 2020). Informed consent was waived.

### Surgical technique

Patients were intubated with a double-lumen endotracheal tube to allow one-lung ventilation during the surgical procedure. The radial artery was used for invasive blood pressure monitoring, with a second arterial line placed in the left femoral artery as a safety net in case of urgent cannulation. Under transesophageal echocardiography in bicaval view, a guide wire was placed in the right femoral vein and advanced to the superior vena cava for venous cannulation. The right hemithorax was elevated to improve surgical access for the mini-thoracotomy. A 5 cm skin incision was performed 2 cm under the fourth intercostal space to access the thoracic cavity obliquely, tunnel-like. In this way, the risk of an incisional hernia may be reduced. The right pleura was opened in one-lung ventilation. Two 10.5 mm trocars were placed on the anterior axillary line's fourth and sixth intercostal space. A soft tissue retractor and a small thoracic retractor were used to spread the ribs and facilitate access to the surgical site. The pericardium was opened approximately three centimeters above the right phrenic nerve with electrocautery. Four pericardial stay sutures were placed, two cranially and two caudally, to obtain appropriate heart exposition. A thoracoscopic camera was put through the fourth intercostal space's trocar, while the sixth intercostal space accommodated a small tube for the insufflation of CO2 in the thoracic cavity and the cardiotomy suction cannula. After heparin administration, the arterial cannulation (Sorin EasyFlow Aortic cannula 23 Fr; LivaNova, London, UK) was performed directly through the ascending aorta and fixed with double purse string sutures. The venous cannulation (Sorin RAP Femoral venous cannula 23/25 Fr; LivaNova, London, UK) was performed in Seldinger-Technique under transesophageal echocardiography through the groin.

A bicaval cannulation with placing an additional cannula in the superior vena cava through the mini-thoracotomy was performed in case of simultaneous tricuspid valve surgery. After establishing the extracorporeal circulation, the aorta was clamped with a flexible aortic clamp, and Bretschneider cardioplegia was delivered directly into the aortic root through a long cannula [Medtronic Aortic Root (Minimally Invasive Aortic Root—MiAR) cannula 9 Fr; Medtronic, Dublin, Ireland]. The left atrium was opened in the Waterston's groove, and the exposition of the mitral valve was enabled through a transthoracic atrial retractor. When reconstruction of the mitral valve was feasible, a ring annuloplasty was applied concomitantly with standard reconstruction techniques, including triangular/quadrangular resection, neo-chordae implantation, and sliding plasty. The ring implantation was always performed with a semi-rigid ring (LivaNova Memo 3D/Rechord and Memo 4D; LivaNova, London, UK) to improve annulus stability without reducing its natural flexibility. In the case of mitral valve stenosis, a valve replacement was executed, preserving the anterior and posterior leaflet. A quadrangular resection of the A2 segment was performed in all patients undergoing mitral valve replacement to reduce the risk of a systolic anterior motion. Pulmonary vein isolation was also performed with concomitant closure of the left atrial appendage or closure of persistent foramen ovale.

### Statistical analysis

Categorical variables are presented as counts (percentages), continuous variables as mean ± standard deviation by customarily distributed data and median (first and third quartile) by non-normally distributed data. Kaplan–Meier survival curves with 95% confidence intervals (95% CI) were used to analyze and plot time-related endpoints. Categorical data were compared with Fisher's exact test because the expected count was less than 5 in some cells of the respective contingency tables. Multiple logistic regression analysis was used for the adverse event outcome, which was a composite outcome consisting of either failed mitral valve repair with early reoperation, reexploration for bleeding, low cardiac output syndrome, postoperative stroke, postoperative myocardial infarction, postoperative new dialysis or ICU stay longer than three days. Univariable and stepwise multivariable logistic regression (both forward and backward) analyses were performed, and the results are reported as odds ratios (OR) with 95% CI and p-values. After stepwise regression modeling, six out of eighteen variables were discarded. The remaining twelve variables included age, sex, body mass index > 30 kg/m^2^, preexisting chronic obstructive pulmonary disease (COPD), preexisting cerebrovascular accident, preexisting active endocarditis, preexisting atrial fibrillation, preexisting cardiac intervention, preexisting cardiac surgery, concomitant tricuspid valve surgery, concomitant ablation, concomitant left atrial appendage closure. All analyses were carried out with R version 4.0.2 (2020-06-22) (R Core Team (2020)). R Markdown was used for dynamic reporting. Distribution over time of perioperative complications, defined as clinical endpoints VARC-2 consensus document and CUSUM and Shewhart charts for assessment of cardiopulmonary bypass and aortic cross-clamping duration over time, have been performed for learning curve assessment. For CUSUM and Shewhart charts, the R package "qcc" was used. Settings for CUSUM charts included: Target: center of group statistics, Decision interval (std. err): 5 and shift detection (std. err): 1. Settings for Shewhart charts included: Target: center of group statistics and upper and lower control limit: ± 3σ from the center of the group statistics.

## Results

### Demographic data

The baseline characteristics of our patient cohort are summarized in Table 1. The vast majority of patients presented with severe mitral valve regurgitation (N = 255; 95.8%), whereas only 4.1% presented with mitral valve stenosis (N = 11). The etiology of mitral valve regurgitation included isolated or bi-leaflet prolapse in 85%, mitral annulus dilatation in 18%, and active mitral valve endocarditis in 6%. The etiology of mitral valve stenosis included degenerative disorder and post-rheumatic leaflet changes in 85% and 15%, respectively. The mean patient age was 67 ± 13 years, and the mean preoperative left ventricular ejection fraction was 59 ± 8.7%. Seven patients (2.6%) had previous cardiac surgery via median sternotomy, whereas 3 patients (1.1%) underwent previous transcatheter edge-to-edge-repair. Coronary artery disease could be excluded preoperatively in all patients via coronary angiography or heart computed tomography, the latter performed primarily in younger patients (< 40 years old). Almost 90% (N = 255) of the procedures were performed in an elective setting, with the remaining 10% (N = 11) being performed urgently.

**Table 1:** Baseline characteristics

Variables	Total patient cohort
	*n* = 266
	No. (%)
Age	66.6 ± 12.8
LVEF (mean)	59 ± 8.7
EuroSCORE II	2.5 ± 3.1
Male	187 (70.3)
Preoperative cerebrovascular accidents	12 (4.5)
COPD	12 (4.5)
Endocarditis	16 (6.0)
Sinus rhythm	205 (77.1)
Paroxysmal atrial fibrillation	32 (12.0)
Persistent/permanent atrial fibrillation	29 (10.9)
No previous cardiac intervention	256 (96.2)
Previous surgical intervention	7 (2.6)
Previous TEER	3 (1.1)
Mitral valve regurgitation	255 (95.8)
Mitral valve stenosis	11 (4.1)

COPD = Chronic obstructive pulmonary disease; LVEF = Left ventricular ejection fraction; TEER = Transcatheter edge-to-edge repair

### Overview of surgical procedures

Mitral valve reconstruction was performed in 85% of our patients (N = 226). Forty patients (15%) underwent mitral valve replacement due to the presence of mitral valve stenosis (N = 11), mitral annulus calcification (N = 10), previous Mitraclip® intervention (N = 3), and endocarditis (N = 14). In 2 cases, mitral valve replacement was performed in a second pump run after unsuccessful mitral valve repair. For the precise calculation of the reconstruction rate in our series, patients presented with mitral valve stenosis (N = 11) or extensive mitral valve endocarditis (N = 14) with the severe distraction of both mitral leaflets were subtracted from the entire patient cohort (N = 266); thus, mitral valve reconstruction-rate in our series counted 95%. Twenty patients (7.5%) required concomitant tricuspid valve reconstruction. Six patients (2.2%) required a concomitant tricuspid valve replacement. Another 55 patients (20.7%) underwent left atrial ablation, whereas left atrial appendage occlusion was performed in 57 patients (21.4%). Table 2 summarizes all surgical procedures.

**Table 2:** Procedures

Variables	Total patient cohort
	*n* = 266
	No. (%)
Mitral valve repair	226 (84.9)
Ring annuloplasty	226
Artificial chordae	62
Leaflet resection	127
Leaflet plication	23
Commissural adaptation	25
Mitral valve replacement	40 (15)
Tricuspid valve repair	20 (7.5)
Ablation	55 (20.7)
ASD closure	42 (15.8)
Left atrial appendage closure	57 (21.4)

ASD = Atrial septal defect

### Perioperative mortality and morbidity

There was no intraoperative mortality in our series. Three procedures (1.1%) had to be converted to median sternotomy, one due to intramural hematoma of the ascending aorta following arterial cannulation and two due to atrial bleeding. The median cross-clamp time was 71.6 (46.6–93.4) minutes, and the median cardiopulmonary bypass time was 93.5 (77–115) minutes. Thirty-day mortality was 1.5% (N = 4). Two patients died due to sepsis and consecutive multiorgan failure, and two were due to vast intestinal ischemia and pulmonary embolism. Postoperative neurological complications, including stroke and intracranial bleeding, were detected with an incidence of 1.1% (N = 3).

Further, postoperative complications consisted of re-exploration for bleeding in 2.6% (N = 7) and myocardial infarction in 1.1% (N = 3) of the patients. Two out of seven patients required reexploration due to bleeding from the left atrial accessway. Three further patients were reoperated due to bleeding from the right thoracic wall, and two other patients due to coagulopathy. Implantation of a permanent pacemaker due to grade III atrioventricular block or bradyarrhythmia was necessary for 4 patients (1.5%) postoperatively. Table 3 summarizes the perioperative data and outcomes of our patient cohort.

**Table 3:** Perioperative data and outcomes

Variables	Total patient cohort	Isolated MV procedures	MV + Concomitant procedures
	No. (%)	No. (%)	No. (%)
*Operation status*			
Elective surgery	237(89.1)	159 (86)	78 (96)
Urgent surgery	21(7.9)	19 (10)	2. (2.5)
Emergency surgery	8(3)	7 (4)	1 (1.5)
Surgical access way			
Right mini-thoracotomy	266 (100)	185 (100)	81. (100)
*Outcome*			
Cross-clamp time (min)	71.6 (46.6–93.4)	59.2	75.7
CPB time (min)	93.5 (77–115)	87.4	106.8
Conversion to median sternotomy	3(1.1)	2 (1.1)	1 (1.5)
Reexploration for bleeding	7(2.6)	5 (2.7)	2 (2.5)
Stroke permanent/transient	3(1.1)	2 (1.1)	1 (1.5)
Myocardial infarction	3(1.1)	1 (0.5)	2 (2.5)
Permanent Pacemaker implantation	4 (1.5)	1 (0.5)	3 (3.7)
Renal failure requiring dialysis	1(0.4)	0 (0)	1 (1.5)
Wound infection	0(0)	0. (0)	0 (0)
Thirty-day Mortality	4(1.5)	2 (1.1)	2 (2.5)
Median ICU-Stay (h)	20 (8.5–42.3)	17	26

CPB = Cardiopulmonary bypass; ICU = Intensive care Unit; MV = Mitral valve

### Midterm clinical outcomes

**Fig. 1:**
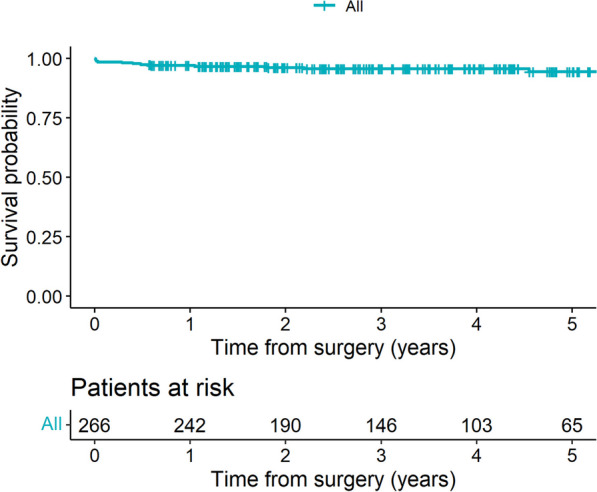
Kaplan- Meier analysis revealing overall survival of 96.56 ± 1.2% at 3 years follow-up

The mean clinical follow-up was 37 ± 22 months. Clinical follow-up was 100% complete. During follow-up, 9 patients (mean age 79 ± 8.5 years) of our cohort died. Causes of late mortality were septic shock following ileus (N = 1), pneumonia (N = 3), and malignancy (N = 1). Cardiac-related midterm mortality has been detected in 1 patient who died following myocardial infarction and heart failure. In 3 patients, the precise cause of death could not be seen. Based on Kaplan–Meier analysis (Fig. 1), overall survival was 96.56 ± 1.2% at 3 years. Only one (0.4%) major neurological event (bleeding) occurred during follow-up.

### Valve-related complications and echocardiographic follow-up

Four patients required reoperation on the index valve early postoperatively due to annuloplasty-ring dehiscence, both taking place in the posteromedial commissure (N = 2) and tear out of anterior leaflet neo-chordae (N = 2). All reoperations were performed via median sternotomy. All patients, except one, underwent repeat mitral valve repair. Dehiscent annuloplasty rings were refixed using single Teflon-reinforced stitches, enabling atrial tissue to be used for better stability. In one case, the mitral valve could be re-reconstructed following the tear out of neo-chordae placed on the anterior mitral leaflet during the initial procedure. In that case, Edge-to-Edge repair using Teflon reinforced 5/0 Prolene stitch enabled the repeated reconstruction of the mitral valve. In the last redo procedure, mitral valve replacement was performed once a tear out of the neo-chordae placed on the anterior mitral leaflet caused a transversal tear on A2-Segment. Thus, the actuarial reoperation-free rate at the index valve was 98.5%.

The mean echocardiographic follow-up time was 27 ± 21 months and 97% complete. At the latest follow-up, 166 patients (67%) showed no or trivial mitral valve regurgitation, 71 patients (29%) had mitral valve regurgitation grade 1 + , 7 (3%) mitral valve regurgitation grade 2, + and 3 patients (1.2%) presented with mitral valve regurgitation grade 3 + . Mean and peak postoperative transvalvular gradient was low [dp mean = 2.5 (2–3) mmHg; dp peak = 7 (5–10) mmHg] and did not increase during follow-up (p_mean_ = 0.146; p_peak_ = 0.211).

### Learning curve and multiple regression analyses for adverse event outcomes

**Fig. 2:**
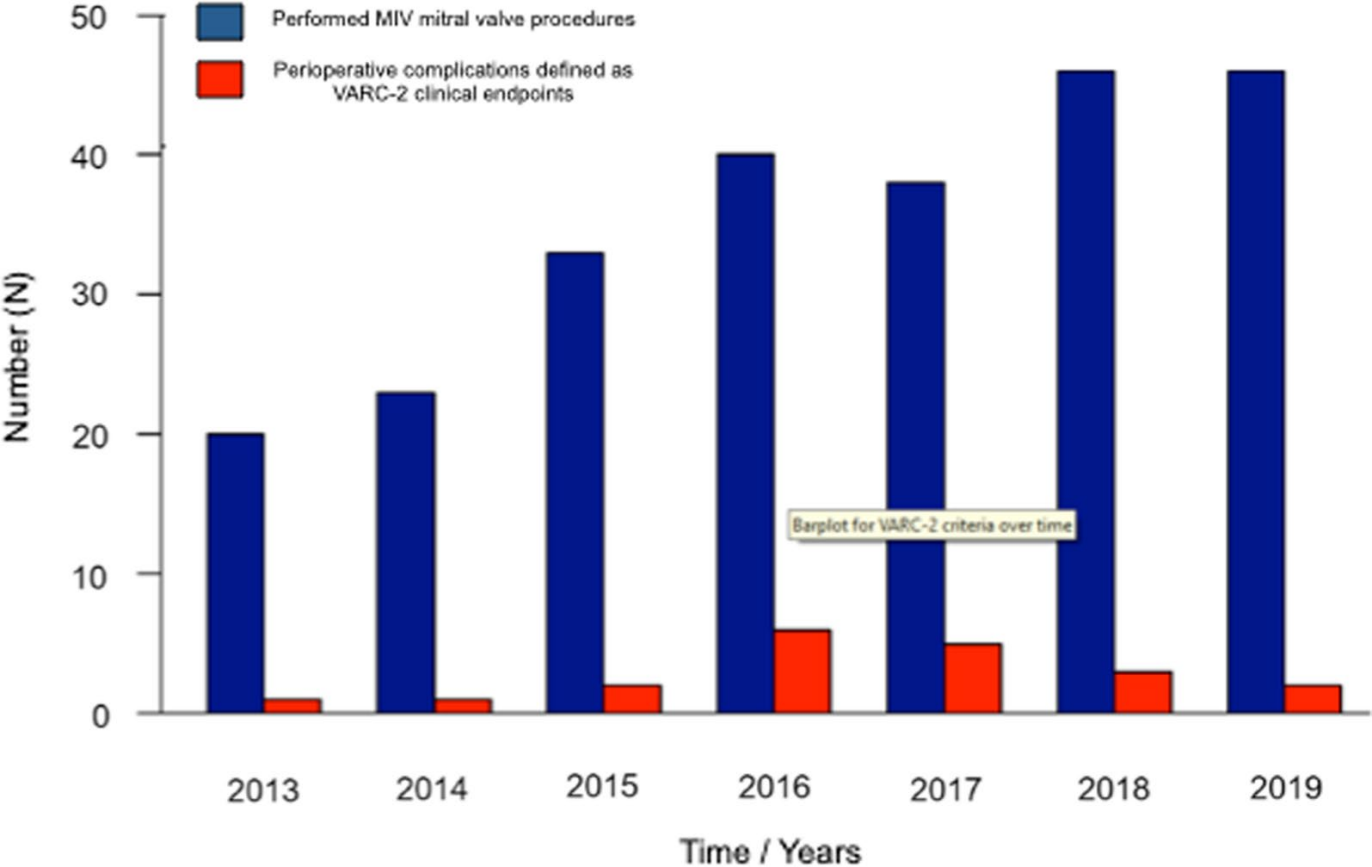
Assessment of our learning curve by evaluation of the rate of perioperative complications defined as Valve Academic Research Consortium-2 (VARC-2) clinical endpoints. The number of minimally invasive (MIV) mitral valve procedures performed per year is shown in blue bars, and the number of perioperative complications defined as VARC-2 clinical points per years is shown in red bars. VARC-2 clinical endpoints include death, stroke, myocardial infarction, bleeding, vascular complications, acute kidney injury, conduction disturbances and valve related complications

For a better assessment of our learning curve distribution of perioperative complications over time, defined as clinical endpoints in the Valve Academic Research Consortium-2 consensus document (VARC-2), including death, stroke, myocardial infarction, bleeding and vascular complications, acute kidney injury, conduction disturbances, and valve-related complications were analyzed. As depicted in Fig. 2, the trend is towards an improved complication rate after the initial 100 procedures.

**Fig. 3:**
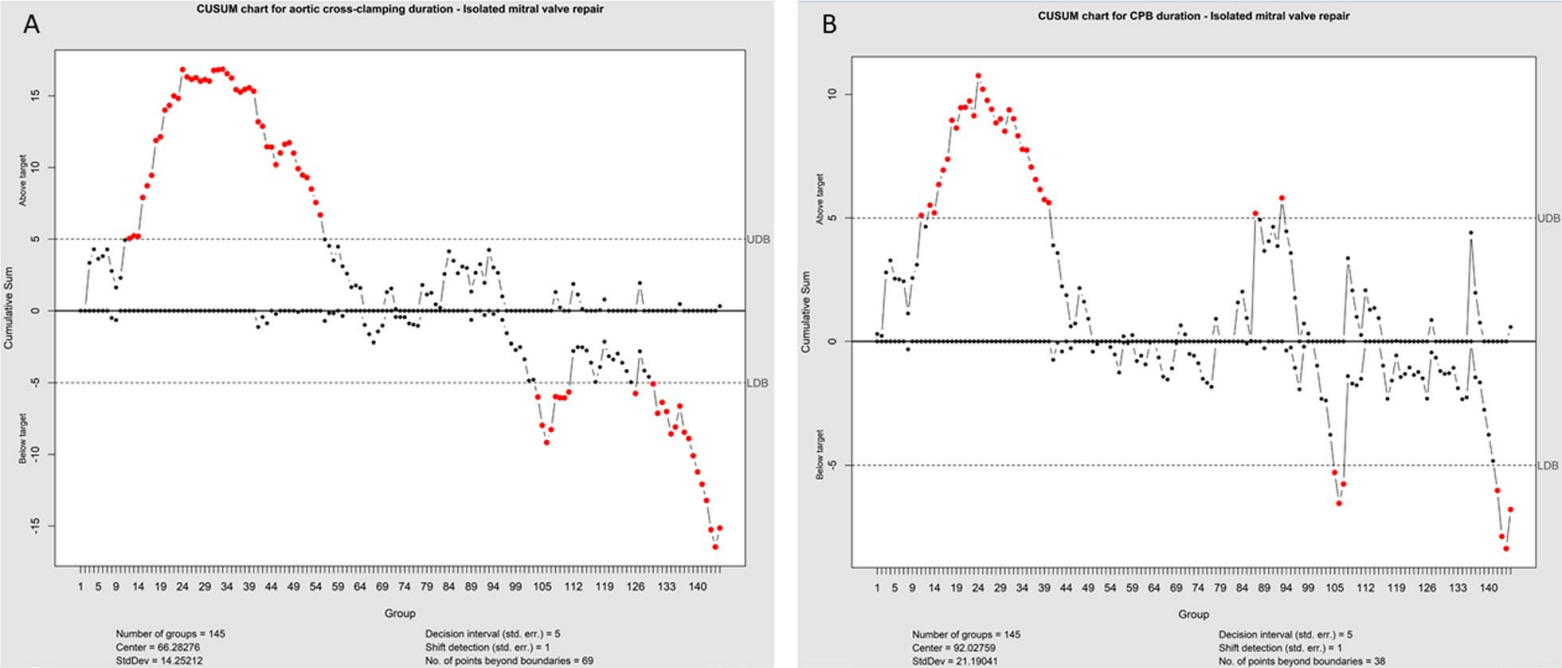
CUSUM chart for aortic cross-clamping time (**A**) in isolated mitral valve repair reveals higher cross-clamping times (CUSUM curve above upper decision limit) for cases 12–55 (year 2013–2016). Afterward (cases 56–103) reduction towards the center of group statistics with lower cross-clamping times (CUSUM curve below lower decision limit) for cases 104–111 (year 2018), case 126 (year 2019), and cases 130–145 (year 2019) was detected. Higher CPB times were seen for mitral valve repair (**B**) for cases 11–40. Afterward, there was a reduction towards the group's center with lower CBP times for cases 105–107 and 142–145

**Fig. 4:**
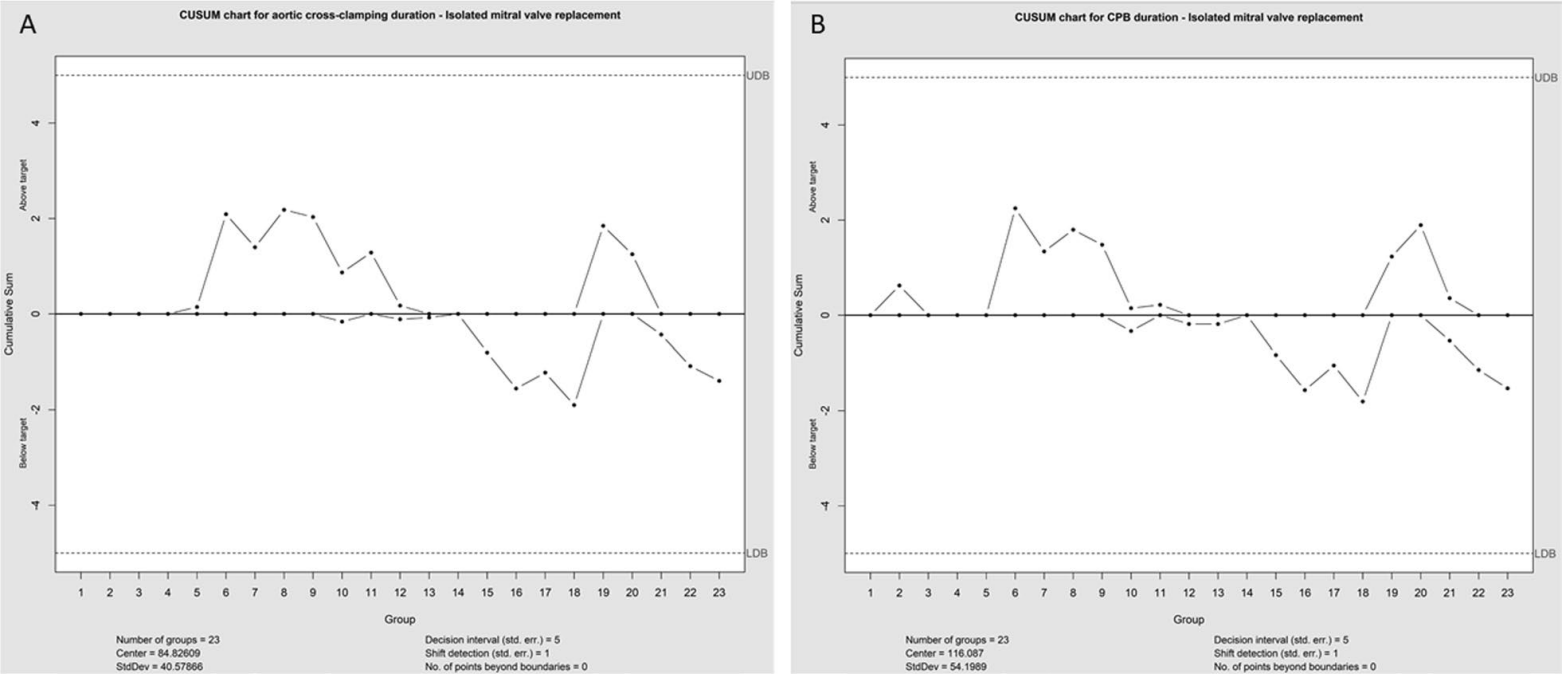
CUSUM charts for aortic cross-clamping and CBP-time in isolated mitral valve replacement illustrate all values within upper and lower detection limits as depicted in **A** and **B**

CUSUM chart for aortic cross-clamping time in isolated mitral valve repair revealed longer cross-clamping times (CUSUM curve above upper decision limit) for cases 12–55 (2013–2016). Afterward (cases 56–103) reduction towards the center of group statistics and finally shorter cross-clamping times (CUSUM curve below lower decision limit) for cases 104–111 (year 2018), case 126 (year 2019), and cases 130–145 (year 2019) were detected. Figure 3 illustrates CUSUM charts for aortic clamping duration (A) and CBP duration (B) in isolated mitral valve repair cases. CUSUM charts for aortic cross-clamping and CBP-time in isolated mitral valve replacement illustrate all values within upper and lower decision limits as depicted in Fig. 4A and B.

Multivariable analysis revealed that concomitant tricuspid valve surgery (OR: 4.44; 95%CI: 1.61–11.80; *p* = 0.003) was significantly associated with adverse event outcomes. Despite a trend towards adverse event outcomes in patients with preexisting active mitral valve endocarditis (OR: 2.69; 95%CI: 0.81–7.87; *p* = 0.082), mitral valve pathology did not significantly affect postoperative morbidity and mortality. Significant findings of the univariable and multivariable analysis are summarized in Table 4.

**Table 4:** Multivariable analysis revealed that concomitant tricuspid valve surgery was significantly associated with adverse event outcomes

Dependent	OR (95%CI)	OR (95%CI)	*p*-value
Composite outcome	Univariable	Multivariable	Uni./Multi
BMI > 30 (Kg/m^2^)	1.61 (0.61–3.92)	1.81 (0.66–4.55)	0.289/0.223
COPD	0.48 (0.03–2.59)	0.44 (0.02–2.46)	0.493/0.444
Active endocarditis	2.69 (0.81–7.87)	3.19 (0.93–9.76)	0.082/0.049
Conc. Tric. Valve Repair	4.28 (1.57–11.17)	4.44 (1.61–11.80)	0.003/0.003

Despite a trend toward adverse event outcomes in patients with preexisting active mitral valve endocarditis, mitral valve pathology did not considerably impact postoperative morbidity and mortality

BMI = Body mass index; COPD = Chronic obstructive pulmonary disease; Conc. = Concomitant; Multi. = Multivariable; Tric. = Tricuspid; Uni. = Univariable

## Discussion

In the past decades, right mini-thoracotomy became a standard mitral valve repair and replacement approach in many centers [7–9]. Durable long-term results, reduced length of stay, and cost-effectiveness led many surgeons to adopt minimally invasive mitral valve surgery into their practice [8, 9]. In 2013, we started using the right mini-thoracotomy for mitral valve surgery in our institution. In the current manuscript, we aim to report our experience and lessons learned from implementing mitral valve surgery through a right mini-thoracotomy in the modern era of MIV-MVS, assessing our learning curve under evaluation of clinical and echocardiographic midterm follow-up data. Furthermore, to the best of our knowledge, there is a lack of evidence regarding risk factors associated with adverse outcomes during the establishment of minimally invasive mitral valve surgery. Thus, risk factor analysis for adverse outcomes in our series has been additionally performed.

All of the procedures were performed by a single leading surgeon with significant experience in mitral valve surgery via sternotomy. In the first year of our experience (2013), mitral valve endocarditis and urgent or emergency mitral valve procedures were considered contraindications for a minimally invasive approach. Since 2014, all mitral valve procedures independent of the underlying pathology or urgency were performed via lateral mini-thoracotomy. Contraindications for the performance of minimally invasive accessway in our series were pectus excavatum, previous surgical procedures in the right thoracic cavity, and concomitant moderate to severe aortic valve regurgitation. Overall incidences of new-onset postoperative stroke, myocardial infarction, renal failure requiring dialysis, and thirty-day mortality were low and comparable with other series published in the past [10–16]. The requirement of repeat thoracotomy for bleeding may represent a drawback of minimally invasive mitral valve surgery because the entire operative field cannot be directly visualized [3]. Our observed incidence of re-thoracotomy for bleeding was 2.6%, comparable with re-sternotomy rates following mitral valve surgery through a median sternotomy (1–4.5%) [17–22]. Thus, based on our findings, the right mini-thoracotomy represents a safe, minimally invasive access for mitral valve surgery, as highlighted in our low early mortality and morbidity.

The main concern in that field remains the risk of a learning curve. The number of patients needed to overcome a substantial learning curve and initial patient selection is still controversially discussed in the literature [5, 6]. There are many tools to analyze the learning curve in minimally invasive surgery. CUSUM represents a frequently applied method helping track early changes in cardiopulmonary bypass and cross-clamp duration over time [3, 23]. The reported results in that field largely varied among authors. Wu and associates analyzed their experience with the first 100 patients undergoing mitral valve surgery via right mini-thoracotomy in 2019 [5]. According to their findings, the number of procedures required to overcome the learning curve was 33 procedures. Holzhey and associates evaluated the individual learning curve of 17 surgeons performing their first surgery in a high-volume center [3]. In a series of 3895 minimally invasively performed mitral valve procedures over 17 years, they detected a learning curve with a turning point toward a lower complication rate after 75 to 125 procedures. These findings could be confirmed by Vo et al. in a series of 204 cases [23]. A further interesting result in their series was the lower volume of mitral valve replacement procedures (60 cases) needed for an acceptable technical complication rate. Our series CUSUM chart for the aortic cross-clamping time in isolated mitral valve repair revealed 55 cases required to overcome the learning curve. Similar to Vo et al. The CUSUM chart for the aortic cross-clamping time in our series demonstrated a lower number of cases required to overcome the learning curve in the field of mitral valve replacement [23].

To give a better concept, we assessed our learning curve additionally by analyzing the distribution of perioperative complications over time, defined as clinical endpoints in the Valve Academic Research Consortium-2 consensus document (VARC-2), including death, stroke, myocardial infarction, bleeding, and vascular complications, acute kidney injury, conduction disturbances, and valve-related complications were analyzed. The current study demonstrates a trend toward reducing complication rates defined as clinical endpoints in the Valve Academic Research Consortium-2 consensus document (VARC-2) over time [24]. Our results align with Holzey et al. and Vo et al., as 65 to 100 cases were necessary to overcome the VARC-2 assessed learning curve [3, 23].

Besides the number of operations, patient selection is crucial for safely implementing a minimally invasive mitral surgery program. Niessen and associates emphasized in a review article that considering those patients with isolated mitral valve disease, avoiding the need for multiple procedures because early CPB and cross-clamp times will be longer was critical while building a new minimally invasive valve surgery program [24, 25]. In our series, careful evaluation of risk factors associated with adverse outcomes during establishing a minimally invasive mitral valve surgery program has been performed. Multivariable analysis revealed that concomitant tricuspid valve surgery was significantly (*p* = 0.003) associated with adverse event outcomes. Furthermore, a trend toward adverse event outcomes in patients with preexisting active mitral valve endocarditis (*p* = 0.082) could be detected. Thus, according to our findings, ideal situations for initial experience in minimally invasive mitral surgery include valve surgery in the presence of degenerative mitral valve diseases, such as planned repair of focal P2 prolapse or straightforward replacement.

In the present study, some limitations need to be addressed—the retrospective nature and the low number of patients analyzed in the current report.

## Conclusions

In conclusion, looking back on our exciting experience in the field of minimally invasive surgery, we may summarize that mitral valve surgery via right-sided mini-thoracotomy can be performed safely with low rates of perioperative and mid-term morbidity and mortality.

According to our findings, careful patient selection regarding straightforward mitral valve surgery in the presence of degenerative mitral valve disease may represent a further important issue, especially at the initial phase of establishing a minimally invasive mitral valve surgery program.

## Data Availability

The datasets used and analyzed during the current study are available from the corresponding author on reasonable request.

## Abbreviations

CPB: Cardiopulmonary bypass
CUSUM: Cumulativ sum
ICU: Intensive care unit
MIV-MVS: Minimally invasive mitral valve surgery
VARC-2: Valve Academic Research Consortium-2
